# Patterns of Pediatric Chronic Hand Eczema: A Systematic Review With Focus on Causes and Management

**DOI:** 10.1177/12034754251322883

**Published:** 2025-02-26

**Authors:** Katie C. Y. Yeung, Joshua Lowe, Jessica S. S. Ho, Sonja Molin

**Affiliations:** 1 Division of Dermatology, University of Ottawa, Ottawa, ON, Canada; 2 Department of Family Medicine, Queen’s University, Kingston, ON, Canada; 3 Division of Dermatology, McGill University, Montreal, QC, Canada; 4 Division of Dermatology, Queen’s University, Kingston, ON, Canada; 5 Department of Dermatology, Venerology and Allergy, Charité-Universitätsmedizin Berlin, Berlin, Germany

**Keywords:** chronic hand eczema, hand dermatitis, pediatric, children, systematic review

## Abstract

Chronic hand eczema (CHE) is commonly seen in adults and often in the context of occupational exposures. Recently, there has been a growing number of cases reported among children. We conducted a systematic review using the PRISMA framework to identify cases of pediatric CHE. Search terms included “eczema,” “dermatitis,” “pompholyx,” “dyshidrosis,” “contact allergy,” and “pulpitis.” Case reports of patients aged <18 years old without an alternate/confounding diagnosis were included. 62 cases were included with a mean age of 10.9 years. In the patients with reported data, 61% (28/46) had a history of atopy and 38% (14/37) reported lesions were present for >1 year. The most common cause was allergic-contact dermatitis (71%) with the bilateral hands affected (87%). A total of 35 unique triggers were identified, with the top 5 being homemade slime (n = 28), store-bought slime (n = 8), outdoor plants (n = 4), UV-curing methacrylate nail polish (n = 4), and sporting gloves (n = 4). Patch testing was performed in 87% (54/62) of patients, of which 96% (52/54) tested positive to 1+ allergens. Positive reactions to methylchloroisothiazolinone and/or methylisothiazolinone (MCI/MI) were among the most common. In 53% (33/62) of patients, removal of the trigger resulted in resolution. Patterns of CHE triggers in pediatric patients differ from adults, and workup should include a detailed history of leisure-time and school activities. MCI/MI was the most common culprit, and trends involving children making slime has led to an increase in prolonged/repeated exposure. Awareness of potential causes ensures early identification, patch testing, prompt removal of trigger, and appropriate management.

## Introduction

### Background

Chronic hand eczema (CHE) is a common inflammatory disease with a significant impact on quality of life. Historically, CHE has been considered a condition primarily affecting adults in occupations involving wet-work or contact-allergen exposure (eg, food handlers, health care workers, and hairdressers). However, there has been a growing number of pediatric-CHE (P-CHE) cases reported. Recent estimates place the lifetime prevalence of P-CHE at 6.5% to 13.3% and the 1 year prevalence at 5.2% to 10.0%.^1^ Despite the high prevalence and known detrimental effects that P-CHE can have on cognitive and social activities during key developmental periods in childhood, there continues to be a lack of literature on the topic.^2-4^ The absence of published P-CHE guidelines means that providers are left relying on a combination of personal clinical experience, anecdotal evidence, and generalization of adult CHE guidelines to inform their management. These adult guidelines, while valuable, cannot be directly applied to children and within themselves demonstrate substantial differences of agreement among experts. Therefore, highlighting the existing knowledge gap in P-CHE.^5^

### Objectives

This systematic review aimed to summarize patterns in the risk factors and presentation of CHE in pediatric populations, including the proportional frequency of different triggers and the setting of exposure. Secondary outcomes include describing the associated patch test findings and the most common culprit allergens in this population. Current management and prognosis of P-CHE in published reports are further summarized.

## Method

### Search Strategy

The MEDLINE, Embase, and Web of Science databases were searched in August 2022 according to the PRISMA (Preferred Reporting Items for Systematic Reviews and Meta-analyses) guidelines to capture all case reports of P-CHE (see Supplemental Appendix 1a). An additional grey literature search was performed. The search was subsequently repeated prior to publication in March 2024 to capture any new literature. The search returned 6521 unique articles, which were each screened by 2 independent reviewers (KY and JH) according to the predefined inclusion and exclusion criteria.

### Eligibility Criteria

We included case reports of patients aged less than 18 years with P-CHE and excluded all other study types. P-CHE was defined as an explicit clinical diagnosis of CHE or hand eczema lasting more than 3 months or occurring twice or more within a year. Cases where the morphology and distribution mimicked CHE, but the authors explicitly came to an alternative diagnosis (eg, peeling skin syndrome) were excluded. Additionally, we excluded patients who had a concurrent confounding diagnosis (eg, diffuse active atopic dermatitis (AD) flare, which also involved the hands). Occupational P-CHE through employment was excluded from this study. There were no language, date, or country restrictions.

### Study Selection and Extraction

In total, 451 full-text articles were assessed for eligibility with each article reviewed by 2 independent reviewers (KY, JH, or JL). Conflicts between reviewers were resolved through consulting a third reviewer. Data were extracted from the included articles using a structured format. The data extracted included the study type, country of publication, patient characteristics and demographics, P-CHE distribution and duration, triggers, setting of exposure, patch test findings where applicable, treatment type, and treatment outcomes.

## Results

### Study and Patient Characteristics

A total of 6521 unique articles were identified through the defined literature search strategy. Following screening and full-text review, 47 articles were included for extraction and analysis including case reports (38/47) and case series (9/47). The included studies represented a total of 62 patients with P-CHE (mean age: 10.9 years, range: 10 months-17 years). Females made up 76% (47/62) of the patients, while males represented 24% (15/62) (see Table S1). In the patients with reported data, 61% (28/46) had a history of atopy—including AD, allergic rhinitis, and/or asthma—compared with 39% (18/46) of cases where there was no known atopic history. Of those with known atopy, 75% (21/28) had a previous diagnosis of AD. Family history of atopy was reported in 15% (9/62) and was unspecified in 81% (50/62).

### Lesion Distribution

Among the 62 children with P-CHE, 87% (54/62) were reported to have lesions involving the bilateral hands while only 13% (8/62) had unilateral involvement. In children with unilateral P-CHE, the finger and/or fingertips were involved in 75% (6/8) of cases. Of the 69% (43/62) of cases where the specific region of the hand that was affected was specified, the palmar surface was involved in 49% (21/43), dorsal surface in 12% (5/43), and both palmar and dorsal surface in 14% (6/43). Involvement of the fingers was specified in 47% (29/62) of all cases, of which the fingertips and/or periungual region were affected in 38% (11/29) (see Table S2).

### Duration of P-CHE

The duration of P-CHE was specified in 60% (37/62) of patients, 13% (8/62) patients were classified as having a recalcitrant or recurrent condition, and in 27% (17/62) of patients, a diagnosis of P-CHE was made by the original author, but the exact duration was unspecified. In children with a specified duration of CHE, 24% (9/37) had lesions present between 3 and 6 months in duration, 38% (14/37) had lesions present for 6 months to 1 year, and 38% (14/37) had lesions present for greater than a year.

### Setting of Exposure

A total of 35 unique triggers were identified and categorized according to the setting of exposure. In 65% (40/62) of cases, the child was exposed to the trigger that led to the development of P-CHE through leisure-time activities. This was defined as play-time activities that were not conducted as part of any school requirements or extracurricular hobbies (such as sports, the arts, or any other routinely scheduled activities) (see Table S3). This was followed by self-care and beauty products (13%), plants (11%), medical and health (10%), sports (8%), clothing and accessories (6%), school and hobbies (6%), and musical activities (3%). Cases where the exposure leading to P-CHE occurred in an occupational setting were excluded.

### Triggers

In 85% (53/62) of patients, there was only 1 identified trigger reported to have led to the development of P-CHE. The remainder of cases were attributed to 2 (11%; 7/62) or 3 concurrent triggers (3%; 2/62). When multiple triggers were present, the patient may be reacting to the same allergen but from multiple different sources, or have more than 1 culprit allergen. The top 4 triggers were as follows: homemade slime (44%), store-bought slime (13%), outdoor plants (6%), and ultraviolet-curing methacrylate nail polish (6%). Repeated exposure to slime through play represented a majority of cases, and homemade slime was a more common culprit than store-bought slime with over 3 times the number of reported cases.

### Patch Testing

Allergic-contact dermatitis (ACD) made up 71% (44/62) of cases. A mixed, irritant, and allergic-contact dermatitis picture was reported in 15% (9/62) of cases, often with 1 preceding the other respectively. Patch testing was performed in 87% (54/62) of patients with P-CHE, of which 96% (52/54) tested positive to 1 or more allergens. Of those that were patch-tested, positive reactions to MCI/MI or MI, were among the most common at 33% (18/54), followed by Sesquiterpene lactone mix 11% (6/54), and Compositae mix 9% (5/54) (see Table S3).

### Chronic Slime P-CHE

**Figure 1.**
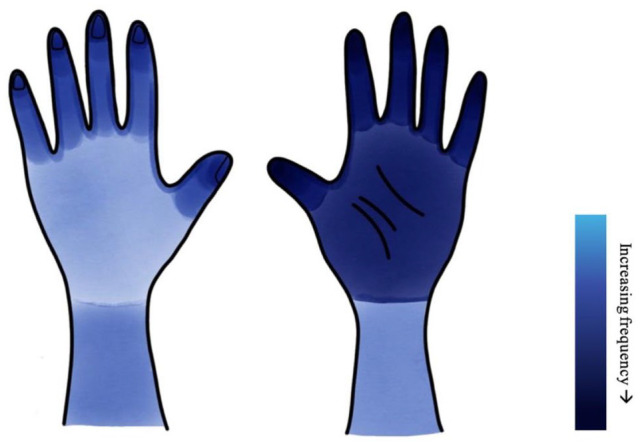
Distribution of lesion location from slime dermatitis.

A total of 45% (28/62) of patients with chronic slime dermatitis affecting the hands were included, representing the greatest proportion of all triggers. Females were overwhelmingly affected representing 93% (26/28) of slime dermatitis cases. The mean age of patients with chronic slime dermatitis was 10.3 years of age, 0.6 years below the overall mean of patients with all-cause P-CHE. Frequency peaked at ages 10 to 12 years with 71% (20/28) of patients falling within this range, compared with 25% (7/28) aged 7 to 9 years, and 4% (1/28) of children aged 13 years or older. Of the 28 children reported to have slime dermatitis, lesion distribution was bilateral in 96% (27/28) of cases. 68% of slime cases (19/28) specified the location of lesions, of which the palmar surfaces of the hands were most commonly implicated. Figure 1 illustrates the distribution of lesions attributed to slime dermatitis and the proportional frequency by colour. Patch testing was performed and reported in 82% (23/28) of patients, with 91% (21/23) of those tested demonstrating 1 or more positive reactions. The top 4 patch test allergens associated with slime dermatitis were as follows: MCI and/or MI in 70% (16/23), linalool in 17% (4/23), benzisothiazolinone in 17% (4/23), and fragrance mix in 13% (3/23) (see Table S4).

### Plant-Related P-CHE

Plant-related dermatitis was the third most commonly reported cause of P-CHE, accounting for 11% (7/62) of cases. Outdoor plants were most commonly implicated contributing to 57% (4/7) of plant-related dermatitis cases. In 75% (3/4) of these children, exacerbation of P-CHE was noted to worsen over the summer corresponding to when the child was more active outdoors where they had regular contact with Compositae plants. Further history revealed that half of these children lived in a rural setting including a dairy farm and in the countryside. Concurrent facial and flexural dermatitis of sun-exposed areas was observed in 1 child and thought to be related to airborne allergen transmission. All children with P-CHE attributed to outdoor plant exposure had a positive reaction to Compositae mix. In the case of a child who developed Compositae-related P-CHE due to indoor houseplants, a keen interest in botany was reported with the child keeping a large collection of plants in the bedroom. There were otherwise no other reported cases of P-CHE resulting from house plants. Notably, 29% (2/7) of children with P-CHE attributed to plants were believed to have developed it in response to exposure to animal feeds. In both cases, the child had been known to pick dandelions to feed their pet rabbits alongside coming into contact with rabbit food. Sesquiterpene lactone mix 0.1% pet. was a common allergen across all 3 types of plant-related dermatitis exposure.

### Nail Enhancement–Related P-CHE

There were 5 children with nail enhancement–related P-CHE, encompassing 3 different types of nail products that have become increasingly popular and accessible among children and adolescents. These include ultraviolet (UV)-curing methacrylate nail polish in (4/5) and both press-on methyl acrylate nail glue and traditional lacquer nail polish (1/5). All 5 children were female ranging from 11 to 17 years of age. All cases involved the bilateral hands, and the most commonly affected areas include the dorsal fingers/fingertips (3/5) and the periungual region (4/5). Among the 5 cases, 1 had P-CHE present for 9 months, another for over a year, and the remaining children had recalcitrant lesions of unspecified duration. In the 1 case of recalcitrant P-CHE where the authors did report on previously trailed treatments, the use of topical corticosteroids (unspecified potency or duration) did not lead to resolution until complete allergen avoidance was also enforced. Post-inflammatory nail changes that persisted after trigger removal were common. Patch testing was performed in 80% (4/5) of the children, and all demonstrated a positive reaction. The most common positive reaction across all 3 types of nail enhancements was to 2-hydroxyethyl methacrylate (2-HEMA). Of note, the child that was using traditional lacquer nail polish, which does not typically contain acrylates, was simultaneously using press-on nails, which attaches to the natural nail using acrylate-based nail glue. As patients may use press-on nails as an alternative to acrylate polishes, they should be made aware that the glue used to attach them may contain several known allergens including poly-methyl methacrylate (MMA), polyethylene glycol dimethacrylate (EGDMA), 2-bromo-2-nitropropane-1,3-diol/bronopol, 2-tert-butyl-4-methoxyphenol, and ethyl cyanoacrylate (ECA), all of which this patient reacted to.

### Sporting Equipment–Related P-CHE

There were 5 children with P-CHE attributed to contact with sporting equipment, representing 8% (5/62) of all cases. All children were male ranging from 9 to 16 years of age. The most common trigger was the use of sporting gloves including soccer goalkeeper gloves in 40% (2/5) of cases and taekwondo gloves in 20% (1/5). Both an intensive shorter period of exposure and prolonged regular contact were implicated. Concurrent bilateral plantar dermatitis was seen in 1 case where the child was also an active swimmer regularly wearing rubber slippers. Two additional sporting exposures reported include rubber bike grips (1/5) and the use of a rubber basketball (1/5). Across the 5 children with sporting equipment–related P-CHE, bilateral involvement of the palmar surfaces was most reported in 80% (4/5) of cases. All 5 children had positive patch test results, of which reaction to the rubber accelerators mercaptobenzothiazole (MBT) and mercapto mix was most common and seen in 60% (3/5) of cases. Trigger removal with or without topical corticosteroids leads to resolution in 80% (4/5) of cases.

### Musical Instrument Related P-CHE

ACD that developed secondary to musical instrument use was reported in 2 separate cases. Both children had unilateral P-CHE that was present for 1 year and the distribution of lesions corresponded with the child’s handling of their musical instrument. In the case of a child with unilateral P-CHE that was worse on the thumb, which was in direct contact with their violin bow, patch testing revealed an allergy to carbamates. The rubber component of their violin bow was identified as the likely trigger, and the occlusion of the bow rubber with surgical tape led to significant improvement within 2 weeks. In the second case where a child had P-CHE affecting their left hand, which was in frequent contact with their guitar strings over a 5 year period, patch testing revealed allergy to nickel sulfate, cobalt chloride, and chromate. The right hand was spared as it was used to hold the plectrum and not in direct contact with the allergen. Avoidance of the guitar by switching to drums instead led to resolution.

### Medical and Health-Related P-CHE

Children with P-CHE attributed to medical and health-related exposures made up 10% (6/62) of all cases. This includes triggers that were either intentionally applied directly to the skin in order to achieve its function [50% (3/6); such as soaps and antiseptic solutions], iatrogenic [17% (1/6); such as a topical antihistamine cream], or were medical/health equipment that came into contact with the skin during the process of being operated [33% (2/6); such as an insulin pump syringe and toothbrush]. In 83% (5/6) of the cases, bilateral involvement was noted and removal of the trigger led to resolution of P-CHE in all 5. In the 2 cases of P-CHE related to use of chlorhexidine-containing disinfectant, the antiseptic was being applied daily for increased infection-control measures. There were no other case reports of P-CHE related to COVID-19 pandemic infection-control measures captured in this review. P-CHE related to liquid soap use was reported in the case of an atopic 7 year old female who presented with a 2 year history of vesicular hand dermatitis affecting the lateral surfaces of the fingers, webbed spaces, volar wrists, lateral palms, and dorsal hands, suggesting residual soap in the interdigital regions and dorsal surfaces after rinsing with water. Patch testing was performed, which revealed an allergy to MCI/MI and MI and 1 month of allergen avoidance led to significant improvement. Notably, P-CHE secondary to topical antihistamine cream was reported in the case of a child prescribed the cream for the treatment of preexisting P-CHE itself, highlighting the importance of reassessing patients with recalcitrant P-CHE to identify any new triggers, including medicaments.

### Presence of Other Lesions

The presence or absence of dermatitis at secondary sites other than the hands was reported for 22 children, of which 59% (13/22) were noted to have involvement. The distribution of lesions reported in addition to concurrent hand eczema included the following: the face (5/13), trunk (3/13), forearms/arms (3/13), flexural surfaces (3/13), feet (2/13), lips (1/13), and insulin pump infusion site (1/13). All cases with secondary sites demonstrated positive findings upon patch testing, supporting an ACD etiology. The trigger most associated with secondary involvement was homemade slime (3/13), with all 3 children experiencing concurrent involvement of the face.

### Management

In 53% (33/62) of patients, removal of the trigger resulted in resolution of P-CHE. Of note, the outcome after trigger removal was not reported in 47% (29/62) of patients. The use of pharmacological therapies was also not well documented, with only 45% (28/62) reported to have received pharmacological therapy, and in 55% (34/62) of cases, the use of medications was unclear. Of those that were reported to have received pharmacological therapy, topical corticosteroids were the most commonly prescribed at 93% (26/28). Notably, 14% (12/28) of patients receiving medications received oral steroids and 18% (5/28) were given oral antibiotics.

### Evidence of Incomplete Ingredient List

In 39% (24/62) of cases, the original authors had noted difficulty in ascertaining the exact composition of the triggers causing P-CHE. The most commonly implicated trigger was slime, representing 79% (19/24) of these cases. In 8% (5/62) of cases, the authors attempted to obtain the complete ingredient list but were impeded by either the ingredients being considered proprietary information or the manufacturer not responding despite attempts to contact them for clarification.

## Discussion

This systematic review summarizes the current literature findings pertaining to the causes of P-CHE, patterns in presentation, patch testing, and the relevant management. A total of 47 studies involving 62 patients were included in this review with a mean age of 10.9 years. In the patients with reported data, 39% of patients did not have a known history of atopy, suggesting the importance of considering allergic P-CHE in the differential diagnosis for all children. Possessing a strong understanding of the most common triggers, settings of exposures, and allergens associated with P-CHE enables clinicians to focus their approach to history-taking and identify the most likely allergen or irritant.

P-CHE may affect children of all ages, with the youngest in the review being just 10 months of age. As young children may be less likely to recall exposures to possible triggers, clinicians need to be familiar with the common culprits to help guide history-taking. Moreover, children often spend extended periods of time away from home such as at school; therefore, knowing which questions to ask parents/teachers/coaches is crucial for promptly identifying the trigger. Delay in diagnosis can lead to severe pruritus and pain, significantly affecting the patient’s life for months to years. Of the 37 patients in whom the exact duration of P-CHE was specified, 38% had lesions present for greater than a year. This highlights the chronic morbidity associated with this condition, which can lead to serious consequences in children during this period of rapid growth and psychosocial development.

The bilateral hands were involved in 86% of children, and while the pattern of distribution does vary depending on the type of trigger a child was exposed to, the palmar surface was involved in almost half of all cases where hand distribution was specified. This suggests that the manipulation of objects through grasping plays a large role in P-CHE. In the patients with reported data, over half also had concurrent dermatitis at a secondary site with the face most commonly affected. Secondary sites of involvement either gradually spread from the primary hand site or occurred at a distant site via direct contact with the trigger or through the child’s autoinoculation. The most common example of autoinoculation was where a child handling homemade slime touches the suspected allergen in the slime and subsequently touches their face. Examples of direct contact to the trigger include a child with Compositae-related P-CHE developing eczema on the soles of her feet from playing outside barefoot, or a child with rubber accelerator-related P-CHE developing similar lesions on the trunk, arms, and feet after sleeping on a rubber air mattress during a sleepover. Airborne contact was also reported in a child with Compositae-related P-CHE who developed simultaneous flares of eczema affecting the sun-exposed areas when playing in the fields, particularly during the summer months. However, in cases where the secondary lesions affect the expected sites of distribution for AD in this age demographic (eg, the antecubital fossa and other flexural surfaces), it remains difficult to determine whether the eczematous changes are due to an AD flare or can be attributed to contact dermatitis.

Patterns of CHE triggers in pediatric patients differ from adults, and workup should take this into consideration. In 65% of cases, the child was exposed to the trigger that led to development of P-CHE through leisure-time activities. This is in contrast to the close association of CHE with occupational exposures in adults. Therefore, the workup of P-CHE should always include a detailed history of leisure-time, school, daycare, and extracurricular activities. This may include, but is not limited to, sports, musical instruments, hobbies, outdoor activities, dress-up time, specific play-time habits, and artistic pursuits. While this review found sex-related differences in the prevalence of common CHE exposures, it is crucial to consider all activities and hobbies when taking a history for children with CHE, without being restricted by gender stereotypes. Moreover, the home environment may also contribute to P-CHE such as Compositae-related P-CHE being more common in children living in more rural settings, further emphasizing the importance of taking a detailed social and environmental history.

While 85% of children only had 1 identified trigger leading to P-CHE, clinicians should always consider the possibility of multiple contributory allergens or irritants, especially in recalcitrant or severe cases. The most common trigger overall was homemade slime (44%).

Patch testing was performed in 87% of patients with P-CHE, of which 96% tested positive to ≥1 allergens. Positive reactions to MCI/MI or MI was among the most common, followed by Sesquiterpene lactone mix, and Compositae mix. In contrast, just 49.2% of patch-tested children in the 2001 to 2018 North American Contact Dermatitis (NACDG) cohort were found to have a relevant reaction to ≥1 NACDG screening allergens.^6^ While both studies suggest high ACD prevalence in P-CHE, this discrepancy is likely attributed to the effect of both inclusion criteria and publication bias magnified by the case reports. Moreover, the risk of false positives, particularly in children with known atopy, background of AD, or had previously been patch-tested may also have contributed.

Notably, providers are often more hesitant to refer pediatric patients for patch testing, given the compliance concerns in younger children who are physically active, have a lower tolerance for keeping the patches on, as well as the fear of sensitization. While there is a risk of sensitization from patch testing, this is minimal and should not pose a barrier to testing. Careful selection of customized allergen panels using a standardized protocol as guided by history can help further mitigate this risk, although it may lead to a risk of false negatives due to “missed allergens” that were not suspected and therefore not tested. An alternative practical approach could include the development of a P-CHE patch series comprising the most common allergens in P-CHE—much like the existing Australian Paediatric Baseline Series. This would allow for targeted testing as informed by our current understanding of common allergens in P-CHE, and be especially helpful for providers who may not regularly patch-test children.

Although patch-testing is a crucial tool for diagnosing ACD in P-CHE, there are unique challenges specific to pediatrics. Namely, the high prevalence of concomitant AD which is estimated to be up to 20%, the influence of recent immunosuppressive/immunomodulatory AD treatment on validity, risk of inciting a flare-up, possibility of false positives if the child is in the refraction phase of former dermatitis, and risk of prior sensitization are all important factors to consider.^7^ Ultimately, with these considerations in mind, patch testing remains the gold standard diagnostic test and should still be performed in recalcitrant cases or where the exact trigger is unknown, but there is a high clinical suspicion for ACD. The importance of patch-testing children as a safe and inexpensive procedure is similarly supported by the most recent European Academy of Allergy and Clinical Immunology (EAACI) position paper, which considers hand dermatitis itself an indication for patch testing. Though, the need of having an experienced clinician that will be able to accurately interpret the results and attribute relevance to positive findings is again emphasized.^8^

Slime dermatitis is a clinical entity that has been increasingly described in the literature, defined as dermatitis resulting from prolonged or frequent contact with slime. Notably, the earliest case report describing P-CHE from slime identified in this review was published by Gittler et al. in May 2018, suggesting that this is a relatively newer phenomenon in dermatology. While slime dermatitis can present acutely or chronically, it often presents as a mixed irritant contact dermatitis that later progresses to involve allergic-contact dermatitis. Females represented majority of included cases, likely reflecting a significant difference in popularity of this new trend between sexes. While slime recipes may vary in composition, an analysis of 48 different online DIY slime recipes by Anderson et al.^9^ found that propylene glycol, MCI/MI, and fragrances were the most common components of homemade slime. Slime dermatitis caused by homemade slime was reported 3 times as often as store-bought slime, likely due to a lack of regulation on the contents included in the mixture. Children often follow online tutorials recommending the inclusion of household products such as shaving foam, detergent, contact lens solution, fragrances, and glues that were never intended for prolonged skin contact and contain ingredients including preservatives like MCI/MI in high concentrations that otherwise would not be permitted on leave-on products. Patch test results were reported in 82% of slime-dermatitis patients in this review, with 91% of those tested demonstrating 1 or more positive reactions. The top 4 allergens were MCI/MI or MI, linalool, benzisothiazolinone in 17% (4/23), and fragrance mix—results that correspond with the culprit ingredients in slime. Patient and family education is crucial as there may be a misinformed belief that because many of the ingredients used for DIY slime are common household products that are frequently applied to the skin they are safe for children to use. Determining the exact composition of homemade slime in particular was difficult as polyvinyl acetate glue, which is used in most DIY recipes, is considered proprietary information.^9^ In general, store-bought slime produced by a regulated manufacturer is considered a safer alternative; however, it still poses a risk of allergy to the contained preservatives and fragrances.

The use of UV-curing methacrylate nail polish by nonprofessionals has become increasingly popular and affordable, leading to an increase in nail enhancement–related P-CHE primarily due to acrylates in the polish. Home acrylic nail kits equipped with a UV lamp were most commonly used, with all included cases being exposed to the trigger in the home setting as opposed to occupationally or in a professional nail salon. Notably, the periungual region was most commonly affected and in some cases presented with significant inflammation and edema alongside nail dystrophy and onycholysis. These findings suggest that the polish may be incompletely cured or were inappropriately applied to the skin as they were not done in a salon by trained aesthetician. In some children, autoinoculation of the uncured polish when touching their face led to secondary facial dermatitis. In 1 case, worsening of P-CHE led to the child increasing their use of nail polish and stick-on nails to conceal the appearance out of embarrassment of being seen by peers, leading to further exacerbation of the condition. Therefore clinicians must provide patient education on the risks of acrylate allergy and ways to minimize risk such as wiping off polish from the skin and ensuring it has completely cured.

While a secondary objective of this review was to analyze P-CHE secondary to increased handwashing and sanitizer use in response to COVID-19 infection-control measures, only 2 cases were captured. This is in contrast to the recent epidemiological studies published by Simonsen et al., which demonstrated an increase in hand eczema due to increased hand hygiene measures during the pandemic among both preschool and schoolchildren.^1,10^ The lack of studies captured by the search strategy may be attributed to several reasons including the following: (1) The published case reports of hand eczema from hand hygiene were acute irritant reactions and thus did not meet the criteria for chronicity; (2) early intervention during the time of increased health vigilance prevented development into a chronic condition; (3) avoidance of seeking medical care in fear of unnecessarily exposing children to hospitals/clinics for a nonacute concern during the pandemic led to decreased presentation; (4) difficulty accessing nonurgent medical care during the pandemic leading to underdiagnosis; and (5) publication bias during the pandemic favouring life-threatening COVID-19 complications. While there is a limitation, CHE from overzealous hand hygiene is well documented in adult wet-work professions such as health care, and thus, similar presentations are likely to occur in pediatric populations.

Overall, management and outcomes were not well documented as resolution after trigger removal was not reported in almost half of patients. The use of pharmacological therapies was also not well documented, with a total of 55% of cases where the use of medications was unclear. Of the 28 patients in whom pharmacological therapy was reported, topical corticosteroids were the most commonly prescribed. However, 14% of children were given oral steroids, which might have been preventable if P-CHE had been diagnosed earlier. Other medications that were trialed included topical antibiotics, topical calcineurin inhibitors, antifungal agents, scabies treatments, and pine tar with zinc oxide ointment. Although novel therapies such as topical JAK inhibitors or systemic IL-4/13 are now available, these were either not reported or not prescribed in the included cases. Moreover, there were insufficient data on treatment response follow-up and time to resolution to allow for subgroup analyses of specific outcomes and trends. Clinicians being aware of potential causes of P-CHE will allow for early identification, prompt removal of trigger, and appropriate management. Regular emollient use has been shown to offer a protective effect against repeated irritant exposure while also acting as an adjunctive treatment for CHE by improving skin barrier function and should be encouraged as an effective means of prophylaxis and adjunct treatment.^11^

## Supplemental Material

sj-docx-1-cms-10.1177_12034754251322883 – Supplemental material for Patterns of Pediatric Chronic Hand Eczema: A Systematic Review with Focus on Causes and Management Supplemental material, sj-docx-1-cms-10.1177_12034754251322883 for Patterns of Pediatric Chronic Hand Eczema: A Systematic Review with Focus on Causes and Management by Katie C. Y. Yeung, Joshua Lowe, Jessica S. S. Ho and Sonja Molin in Journal of Cutaneous Medicine and Surgery

sj-docx-2-cms-10.1177_12034754251322883 – Supplemental material for Patterns of Pediatric Chronic Hand Eczema: A Systematic Review with Focus on Causes and Management Supplemental material, sj-docx-2-cms-10.1177_12034754251322883 for Patterns of Pediatric Chronic Hand Eczema: A Systematic Review with Focus on Causes and Management by Katie C. Y. Yeung, Joshua Lowe, Jessica S. S. Ho and Sonja Molin in Journal of Cutaneous Medicine and Surgery
