# Supplementary material for: Patterns of Pediatric Chronic Hand Eczema: A Systematic Review With Focus on Causes and Management
Source: J Cutan Med Surg. 2025 Feb 26;29(4):386–93. doi: 10.1177/12034754251322883 (PMC12304492; doi:10.1177/12034754251322883)
Supplement: sj-docx-1-cms-10.1177_12034754251322883 – Supplemental material for Patterns of Pediatric Chronic Hand Eczema: A Systematic Review with Focus on Causes and Management [file sj-docx-1-cms-10.1177_12034754251322883.docx]

**Supplementary Tables**

**Table S1. Patient Demographics n (%)**

| **Sample size** | Total number of studies included | 47 |
| --- | --- | --- |
|  | Total number of cases included | 62 |
| **Sex** | Female | 47 (76) |
|  | Male | 15 (24) |
| **Age (years)** | Mean (range) | 10.9 (0.83-17) |
| **Atopic History** | Yes | 28 (45) |
|  | No | 18 (29) |
|  | NS | 16 (26) |
| **Diagnosis** | Allergic contact dermatitis | 44 (71) |
|  | Mixed allergic and irritant contact dermatitis | 9 (15) |
|  | NS | 9 (15) |
| **Distribution** | Unilateral | 8 (13) |
|  | Bilateral | 54 (87) |
| **Patch testing** | Positive | 52 (84) |
|  | Negative | 2 (3) |
|  | Not performed or NS | 8 (13) |
| **Pharmacological management** | Received | 28 (45) |
|  | NS | 34 (55) |
| *NS: Not specified* | | |

**Table S2. Summary of Case Reports (n=62)**

| **Author, Year** | **Country** | **n** | **Age (years), Sex** | **Atopy** | **Identified Trigger** | **Diagnosis** | **Distribution, Site** | **Patch Test** | **Trigger Removal, Pharmacological Intervention, Resolution** |
| --- | --- | --- | --- | --- | --- | --- | --- | --- | --- |
| Aerts 2018 | Belgium | 2 | 11, F | N | Homemade slime and putty | ICD/ACD | B, Palmar surface | Y, + | Trigger removal, NS, Y |
|  |  |  | 9, M | Y | Playdough | ICD/ACD | B, Hands | Y, + | Trigger removal, NS, Y |
| Aizawa 2018 | Japan | 1 | 16, M | NS | Goalkeeper gloves | ACD | B, Palmar/dorsal surfaces and wrists | Y, + | NS, NS, NS |
| AlipourTehrany 2019 | Switzerland | 2 | 12, F | N | Homemade slime | ACD | B, Palmar surface, palmar finger creases, forearms | Y, + | NS, Topical corticosteroids, NS |
|  |  |  | 11, F | Y | Homemade slime | ACD | B, Hands | Y, + | NS, NS, NS |
| Allen 2010 | United Kingdom | 1 | 11, F | NS | Rubber Air Mattress | ACD | B, Palmar surface | Y, + | Trigger removal, Topical corticosteroids, Y |
| Alves 2020 | Portugal | 1 | 11, F | NS | UV (meth)acrylate nail polish | ACD | B, Dorsal fingers | Y, + | Trigger removal, Topical corticosteroids, Y |
| Anderson 2019 | USA | 1 | 11, F | Y | Homemade slime | ACD | B, Palmar surface, palmar finger creases | Y, + | Trigger removal, Topical corticosteroids, Y |
| Blondeel 2003 | Belgium | 1 | 16, F | N | Hair products | ACD | B, Palmar surface of fingers | Y, + | Trigger removal, NS, Y |
| Chave 2003 | United Kingdom | 1 | 17, F | Y | Textile dye | ACD | B, Palmar/dorsal surfaces | Y, + | Trigger removal, Topical corticosteroids and oral antibiotics, Y |
| Coates 2010 | United Kingdom | 2 | 15, F | Y | House plants | ACD | B, Hands | Y, + | NS, Topical corticosteroids, NS |
|  |  |  | 7, F | Y | Outdoor plants | ACD | B, Hands | Y, + | NS, NS, NS |
| Córdoba 2021 | Spain | 1 | 10, F | N | Homemade slime | ACD | B, Palmar/dorsal surfaces, palmar finger creases | Y, + | Trigger removal, Topical corticosteroids and oral steroids, Y |
| Cusano 1989 | Italy | 1 | 16, F | NS | Topical antihistamine cream | ACD | B, Hands | Y, + | NS, NS, NS |
| Dahlin 2016 | Sweden | 2 | 15, F | NS | UV nail polish | ACD | B, Periungual | Y, + | NS, NS, NS |
|  |  |  | 11, F | NS | UV nail polish | ACD | B, Periungual | Y, + | NS, NS, NS |
| Ducharme 2018 | France | 1 | 7, M | Y | Playdough | ACD | B, Fingertips | Y, + | Trigger removal, topical corticosteroids, Y |
| Emeksiz 2018 | Turkey | 1 | 13, M | NS | Goalkeeper gloves | ACD | B, Palmar surface | Y, + | Trigger removal, NS, Y |
| Flohr 2008 | United Kingdom | 3 | 4, M | Y | Outdoor plants | ACD | B, Palmar/dorsal surfaces and palmar fingers | Y, + | NS, NS, NS |
|  |  |  | 8, M | Y | Outdoor plants | ACD | B, Hands | Y, + | NS, NS, NS |
|  |  |  | 8, F | Y | Outdoor plants | ACD | B, Hands | Y, + | NS, NS, NS |
| Garioch 1988 | United Kingdom | 1 | 17, M | Y | Printer wash | ACD | B, Hands | Y, + | NS, NS, NS |
| Geldof 1989 | Netherlands | 1 | 15, M | NS | Insulin pump syringe | ACD | B, Hands | Y, + | Trigger removal, NS, Y |
| Gittler 2018 | USA | 1 | 9, F | N | Homemade slime | ICD, ACD | B, Palmar surface, periungual, and fingertips | NS | Trigger removal, Topical corticosteroids, topical/oral antibiotics and scabies treatment, Y |
| Gospodinova 2020 | Bulgaria | 1 | 9, F | N | Homemade slime and plasticine | ICD/ACD | B, Palmar surface and periungual | Y, + | Trigger removal, Topical corticosteroids and antibiotics, Y |
| Herro 2011 | USA | 1 | 9, M | Y | Rubber violin bow, tae kwon do gloves, toothbrush grip, and computer mouse | ACD | U, Thumb | Y, + | Trigger removal, NS, Y |
| Husain 1977 | United Kingdom | 1 | 16, F | NS | Nickel coins | NS | U, Palmar fingers | Y, + | Trigger removal, NS, Y |
| Isaksson 2007 | Sweden | 1 | 10, F | Y | Rabbit feed | ACD | B, Palmar surface, fingers, and fingertips | Y, + | Trigger removal, Topical calcineurin inhibitor, Y |
| Jacob 2014 | USA | 1 | 11, M | Y | Macbook pro | ACD | B, Wrists | Y, + | Trigger removal, Topical corticosteroids, Y |
| Kefala 2023 | France | 2 | 16, F | NS | Antiseptic solution | ACD | B, Hands | Y, + | Trigger removal, NS, Y |
|  |  |  | 16, F | NS | Antiseptic solution | ACD | B, Hands | Y, + | Trigger removal, NS, Y |
| Khanna 2016 | USA | 1 | 7, F | Y | Liquid soap | NS | B, Palmar/dorsal surfaces, wrist, fingers, interdigital | Y, + | Trigger removal, Topical corticosteroids, Y |
| Kondratuk 2019 | USA | 2 | 12, F | Y | Homemade slime | NS | U, Dorsal surface and wrist | N | Trigger removal, Topical corticosteroids, topical and oral antibiotics, Y |
|  |  |  | 13, F | NS | Homemade slime | NS | B, Palmar surface | N | NS, NS, NS |
| Kong 2019 | Canada | 1 | 10, F | N | Homemade slime | NS | B, Palmar surface and fingertips | N | NS, NS, NS |
| Lavery 2020 | United Kingdom | 3 | 10, F | Y | Homemade slime | ICD/ACD | B, Hands | Y, + | NS, NS, NS |
|  |  |  | 12, F | N | Homemade slime | ICD/ACD | B, Fingers | Y, - | NS, NS, NS |
|  |  |  | 10, F | N | Homemade slime | ICD/ACD | B, Hands | Y, - | NS, NS, NS |
| Mainwaring 2019 | USA | 1 | 11, F | Y | Homemade slime | ACD | B, Palmar surface and fingers | Y, + | Trigger removal, Topical corticosteroids and oral steroids, Y |
| Morgado 2019 | Portugal | 1 | 11, F | N | Homemade slime | ACD | B, Palmar surface and fingers | Y, + | NS, NS, NS |
| Opalinska 2022 | Poland | 1 | 17, F | NS | UV curing nail polish | ACD | B, Fingertips and periungual | N | Trigger removal, Topical corticosteroids, oral antibiotics and antifungal, Y |
| Ozkaya 2011 | Turkey | 1 | 10, M | N | Rubber bike grip | ACD | B, Palmar surface and interdigital | Y, + | Trigger removal, Topical corticosteroids, Y |
| Papadias-Feria 2019 | USA | 1 | 9, F | Y | Homemade slime | NS | B, Hands and wrists | Y, + | Trigger removal, NS, Y |
| Pereira 2000 | Portugal | 1 | 7, F | NS | Leather gloves | NS | B, Hands | Y, + | NS, NS, NS |
| Pessotti 2020 | Brazil | 1 | 9, F | Y | Homemade slime and putty | ICD/ACD | B, Palmar surface | Y, + | Trigger removal, Oral steroids, Y |
| Piazza 2018 | Brazil | 1 | 11, F | NS | Homemade slime | NS | B, Dorsal fingers | NS | Trigger removal, Topical corticosteroids, Y |
| Quaade 2023 | Denmark | 1 | 13, F | N | Press on nails and traditional lacquer nail polish | ACD | B, Dorsal fingertips and periungual | Y, + | NS, NS, NS |
| Ramoutar 2020 | United Kingdom | 4 | 10, F | Y | Homemade/storebought slime | ACD | B, Hands | Y, + | NS, NS, NS |
|  |  |  | 10, F | N | Homemade/storebought slime and putty | ACD | B, Hands | Y, + | NS, NS, NS |
|  |  |  | 10, F | N | Homemade slime | ACD | B, Hands | Y, + | NS, NS, NS |
|  |  |  | 10, F | Y | Homemade slime | ACD | B, Hands | Y, + | NS, NS, NS |
| Rodriguez-Serna 2002 | USA | 1 | 9, M | Y | Rubber basketball | NS | B, Palmar fingers and thenar eminence | Y, + | Trigger removal, Topical corticosteroids, Y |
| Salman 2019 | Turkey | 3 | 9, F | N | Homemade slime | ACD | B, Periungual and interdigital | Y, + | NS, Topical corticosteroids, NS |
|  |  |  | 12, F | N | Homemade slime | ACD | B, Palmar surface | Y, + | NS, Topical corticosteroids, NS |
|  |  |  | 10, F | N | Homemade slime | ACD | B, Interdigital | Y, + | NS, NS, NS |
| Silverberg 2016 | USA | 1 | 9, F | Y | Metal and rubber costume jewelry, nickel belt buckles and lip balm case | ACD | B, Dorsal surface and periungual | Y, + | NS, Topical corticosteroids, NS |
| Smith 2006 | United Kingdom | 1 | 17, M | Y | Electric guitar | ACD | U, Palmar surface and fingers | Y, + | Trigger removal, NS, Y |
| Vijayasankar 2020 | India | 1 | 0.8, M | N | Cultural wrist thread | ICD/ACD | U, Wrist | NS | NS, Topical corticosteroids, NS |
| Wakelin 1997 | United Kingdom | 1 | 7, M | Y | Rabbit feed | ACD | U, Palmar surface, wrist, interdigital, periungual, fingertips | Y, + | Trigger removal, Topical corticosteroids, oral steroids, and topical/oral antibiotics, Y |
| Walker-Smith 2016 | United Kingdom | 1 | 5, M | Y | Copper coins and ZAMAC alloy cars | ACD | U, Fingertips | Y, + | Trigger removal, Topical corticosteroids and topical calcineurin inhibitor, Y |
| Ward 2017 | USA | 1 | 6, F | Y | Plastic phone case | ACD | U, Palmar surfaces and palmar fingers | N | Trigger removal, Topical corticosteroids, oral antibiotics, and antifungals, Y |
| Zhang 2019 | USA | 1 | 10, F | NS | Homemade slime | ACD | B, Palmar and dorsal fingers | Y, + | Trigger removal, Topical corticosteroids and topical antibiotics, Y |
| ACD: allergic contact dermatitis; B: bilateral; F: female; ICD: irritant contact dermatitis; M: male; N: no; NS: not specified; U: unilateral; Y: yes | | | | | | | | | |

**Table S3. Identified Triggers and Associated Patch Test Findings**

| **Setting of Exposure** | **Identified Trigger** | **Cases (% of total 62 cases)** | **Top 3 associated patch test findings* (% of patients with the identified trigger that tested positive)** |
| --- | --- | --- | --- |
| **Leisure activities** | Homemade slime | 27 (44) | 1. MCI/MI or MI (56) 2. Linalool hydroperoxides; Benzisothiazolinone (BIT) (15) 3. Fragrance mix 1 (7) |
|  | Store-bought slime (including putty, plasticine, playdough) | 8 (13) | 1. MCI/MI or MI (63) 2. Linalool hydroperoxides (50) 3. Benzisothiazolinone (BIT); Fragrance mix 1 (25) |
|  | Rubber sleepover air mattress | 1 (2) | 1. Carba mix; 2-Mercaptobenzothiazole (MBT); Mercapto mix |
|  | Copper coins | 1 (2) | 1. Copper (II) sulfate pentahydrate 2% pet |
|  | Nickel coins | 1 (2) | 1. Nickel sulfate |
|  | ZAMAC Alloy model cars | 1 (2) | 1. Copper (II) sulfate pentahydrate 2% pet |
|  | Plastic phone case | 1 (2) | *­­---* |
|  | Remote control | 1 (2) | 1. Carbamates |
|  | ***Total*** | ***40 (65)*** |  |
| **Self-care and beauty products** | Ultraviolet-curing methacrylate nail polish | 4 (6) | 1. di-Hema trimethylhexyl dicarbamate (di-HEMA); 2-Hydroxyethyl Methacrylate (2-HEMA) (50) 2. Urethane acrylates; MI; 2-hydroxypropyl methacrylate (HPMA); 2-Hydroxyethyl Acrylate (2-HEA); ethyleneglycol dimethacrylate (EGDMA); urethane dimethacrylate (UDMA); methyldibromo glutaronitrile (25) |
|  | Traditional lacquer nail polish | 1 (2) | 1. 2-Hydroxyethyl Methacrylate (2-HEMA); Methyl methacrylate (MMA); Ethyl cyanoacrylate (ECA); Ethyl acrylate; ethyleneglycol dimethacrylate (EGDMA) |
|  | Press-on methyl acrylate nail glue | 1 (2) | 1. 2-Hydroxyethyl Methacrylate (2-HEMA); Methyl methacrylate (MMA); Ethyl cyanoacrylate (ECA); Ethyl acrylate; ethyleneglycol dimethacrylate (EGDMA) |
|  | Hair products | 1 (2) | 1. p-Phenylenediamine (PPD); p-toluenediamine; Ammonium persulfate (APS); decyl glucoside; Lauryl glucoside; Coco glucoside; Cetearyl glucoside |
|  | Nickel lip balm case | 1 (2) | 1. Nickel sulfate; Gold thiosulfate; Thiuram mix |
|  | ***Total*** | ***8 (13)*** |  |
| **Plants** | Outdoor plants | 4 (6) | 1. Compositae mix (100) 2. Sesquiterpene lactone mix 0.1% pet.; Tansy 1%; Arnica 0.5%; Feverfew 1%; Yarrow 1%; Taraxacum 2.5% (75) 3. Chamomile 2.5% (50) |
|  | Animal feed | 2 (3) | 1. Sesquiterpene lactone mix 0.1% pet. (100) 2. Chrysanthemum (10% eth.); Dandelion (10% eth.); Daisy (10% eth.) (50) |
|  | House plants | 1 (2) | 1. Compositae mix; Sesquiterpene lactone mix 0.1% pet. |
|  | ***Total*** | ***7 (11)*** |  |
| **Medical and Health** | Antiseptic solution | 2 (3) | 1. Benzalkonium chloride (100) 2. Chlorhexidine; Benzyl alcohol 5%; Benzyl alcohol 10% (50) |
|  | Insulin pump syringe | 1 (2) | 1. Epoxy resin |
|  | Topical antihistamine cream | 1 (2) | 1. Dexchlorpheniramine maleate; Mepyramine hydrochloride; Diphenhydramine hydrochloride; Pheniramine |
|  | Liquid soap | 1 (2) | 1. MCI/MI; MI |
|  | Toothbrush grip | 1 (2) | 1. Carbamates |
|  | ***Total*** | ***6 (10)*** |  |
| **Sports** | Goalkeeper gloves | 2 (3) | 1. Mercaptobenzothiazole (MBT); Mercapto mix; Carba mix; 1,3-diphenylguanidine; Para-tertiary butylphenol (PTBP) formaldehyde resin (100) |
|  | Tae kwon do gloves | 1 (2) | 1. Carbamates |
|  | Rubber bike grip | 1 (2) | 1. p-Phenylenediamine (PPD); N-Isopropyl-N'-phenyl-p-phenylenediamine (IPPD) |
|  | Rubber basketball | 1 (2) | 1. Mercaptobenzothiazole (MBT); Mercapto mix; Thiuram |
|  | ***Total*** | ***5 (8)*** |  |
| **Clothing and accessories** | Leather gloves | 1 (2) | 1. Potassium dichromate; Cobalt chloride; 4-Tert-butylphenol-formaldehyde resin |
|  | Metal and rubber costume jewelry | 1 (2) | 1. Nickel sulfate; Gold thiosulfate; Thiuram mix |
|  | Nickel belt buckle | 1 (2) | 1. Nickel sulfate; Gold thiosulfate; Thiuram mix |
|  | Cultural wrist thread | 1 (2) | *---* |
|  | ***Total*** | ***4 (6)*** |  |
| **School and Hobbies** | Printer wash | 1 (2) | 1. Fragrance mix 1; Balsam of peru |
|  | Textile dye | 1 (2) | 1. Basic red |
|  | Macbook Pro | 1 (2) | 1. Nickel sulfate |
|  | Computer mouse | 1 (2) | 1. Carbamates |
|  | ***Total*** | ***4 (6)*** |  |
| **Musical activities** | Rubber violin bow | 1 (2) | 1. Carbamates |
|  | Electric guitar | 1 (2) | 1. Nickel sulfate; Cobalt chloride; Chromate |
|  | ***Total*** | ***2 (3)*** |  |
| Total number of unique triggers: 35. Some patients have multiple identified triggers. *Patch test read as + or above on any day. | | | |

**Table S4. Breakdown of the 28 reported slime dermatitis cases n (%)**

| **Sex** | |
| --- | --- |
| Female | 26 (93) |
| Male | 2 (7) |
| **Age (years)** | |
| Mean | 10.3 |
| 7-9 | 7 (25) |
| 10-12 | 20 (71) |
| 13 or above | 1 (4) |
| **Atopy** | |
| History of atopy | 11 (39) |
| Non-atopic | 14 (50) |
| NS | 3 (11) |
| **Type of slime** | |
| Homemade | 27 (96) |
| Store-bought | 8 (29) |
| **Distribution of lesion** | |
| Unilateral | 1 (4) |
| Bilateral | 27 (96) |
| **Patch test** | |
| Positive | 21 (75) |
| Negative | 2 (7) |
| Not performed or NS | 5 (18) |
| **Top 4 most common allergens** | |
| MCI and/or MI | 16 (57) |
| Linalool hydroperoxides | 4 (14) |
| Benzisothiazolinone (BIT) | 4 (14) |
| Fragrance mix 1 | 3 (11) |
| **Total** | **28** |
| *NS: Not specified* | |
