# Supplementary material for: Patterns of Pediatric Chronic Hand Eczema: A Systematic Review With Focus on Causes and Management
Source: J Cutan Med Surg. 2025 Feb 26;29(4):386–93. doi: 10.1177/12034754251322883 (PMC12304492; doi:10.1177/12034754251322883)
Supplement: sj-docx-2-cms-10.1177_12034754251322883 – Supplemental material for Patterns of Pediatric Chronic Hand Eczema: A Systematic Review with Focus on Causes and Management [file sj-docx-2-cms-10.1177_12034754251322883.docx]

**Supplementary Materials**

**Appendix 1a. Search strategy for systematic review**

Database(s): **Ovid MEDLINE ALL** 1946 to March 26 2024 (initial search Aug 27 2022)
Search Strategy:

| # | Searches | Results |
| --- | --- | --- |
| 1 | dermatitis/ or dermatitis, atopic/ or dermatitis, contact/ or dermatitis, allergic contact/ or dermatitis, irritant/ or eczema/ | 74507 |
| 2 | Dermatitis.mp. | 107656 |
| 3 | Dermatos*.mp. | 43481 |
| 4 | Eczema*.mp. | 27776 |
| 5 | Hand dermatoses/ | 8081 |
| 6 | Pompholyx.mp. | 208 |
| 7 | Eczema, Dyshidrotic/ | 306 |
| 8 | Dyshidro*.mp. | 519 |
| 9 | Chronic Urticaria/ | 842 |
| 10 | Chronic urticaria.mp. | 3829 |
| 11 | Chronic hives.mp. | 11 |
| 12 | Contact allergy.mp. | 3461 |
| 13 | Contact sensitivity.mp. | 1519 |
| 14 | Pulpitis/ | 3139 |
| 15 | Pulpitis.mp. | 4230 |
| 16 | 1 or 2 or 3 or 4 or 5 or 6 or 7 or 8 or 9 or 10 or 11 or 12 or 13 or 14 or 15 | 161910 |
| 17 | Hand/ or fingers/ or thumb/ or wrist/ | 94897 |
| 18 | Hand.mp. | 514481 |
| 19 | Hands.mp. | 66753 |
| 20 | Finger.mp. | 99072 |
| 21 | Fingers.mp. | 63612 |
| 22 | Thumb.mp. | 21480 |
| 23 | Wrist.mp. | 53975 |
| 24 | Wrists.mp. | 6023 |
| 25 | Interdigital.mp. | 2761 |
| 26 | Palm*.mp. | 105399 |
| 27 | 17 or 18 or 19 or 20 or 21 or 22 or 23 or 24 or 25 or 26 | 791738 |
| 28 | Child/ or child, preschool/ | 2195921 |
| 29 | Child*.mp. | 2824672 |
| 30 | Infant/ | 877776 |
| 31 | Infant.mp. | 1336454 |
| 32 | Toddler.mp. | 6135 |
| 33 | Adolescent/ | 2239047 |
| 34 | Adolescen*.mp. | 2335273 |
| 35 | Teen*.mp. | 37052 |
| 36 | Students/ | 84191 |
| 37 | Student*.mp. | 418614 |
| 38 | Schools/ | 53347 |
| 39 | School*.mp. | 396917 |
| 40 | Child care/ or infant care/ | 15588 |
| 41 | Child care.mp. | 12445 |
| 42 | Infant care.mp. | 11025 |
| 43 | Kindergarten*.mp. | 8588 |
| 44 | Child day care centers/ or nurseries, infant/ | 6325 |
| 45 | Nursery.mp. | 11963 |
| 46 | Day care.mp. | 15674 |
| 47 | Daycare.mp. | 1938 |
| 48 | Pediatrics/ | 58008 |
| 49 | Pediatr*.mp. | 472300 |
| 50 | Paediatr*.mp. | 90329 |
| 51 | Kid.mp. | 2681 |
| 52 | Kids.mp. | 8700 |
| 53 | 28 or 29 or 30 or 31 or 32 or 33 or 34 or 35 or 36 or 37 or 38 or 39 or 40 or 41 or 42 or 43 or 44 or 45 or 46 or 47 or 48 or 49 or 50 or 51 or 52 | 5059742 |
| 54 | 16 and 27 and 53 | 3309 |
| 55 | limit 54 to (humans and "all child (0 to 18 years)") | 2905 |

Database(s): **Embase Classic+Embase**1947 to 26 March 2024 (initial search Aug 27 2022)
Search Strategy:

| # | Searches | Results |
| --- | --- | --- |
| 1 | dermatitis/ or application site dermatitis/ or application site eczema/ or atopic dermatitis/ or contact dermatitis/ | 128672 |
| 2 | dermatitis.mp. | 179405 |
| 3 | dermatos*.mp. | 38544 |
| 4 | eczema/ or hand eczema/ | 41608 |
| 5 | eczema*.mp. | 64127 |
| 6 | Pompholyx/ | 735 |
| 7 | Pompholyx.mp. | 836 |
| 8 | Dyhidrosis/ | 409 |
| 9 | Dyhidro*.mp. | 869 |
| 10 | Contact allergy/ | 9369 |
| 11 | Contact urticaria/ | 1566 |
| 12 | Contact allergy.mp. | 11437 |
| 13 | Contact sensitivity.mp. | 2483 |
| 14 | Pulpitis/ | 4104 |
| 15 | Pulpitis.mp. | 4723 |
| 16 | Allergic urticaria/ or chronic urticaria/ or contact urticaria/ | 8976 |
| 17 | Urticaria.mp. | 62342 |
| 18 | Hives.mp. | 3063 |
| 19 | 1 or 2 or 3 or 4 or 5 or 6 or 7 or 8 or 9 or 10 or 11 or 12 or 13 or 14 or 15 or 16 or 17 or 18 | 298911 |
| 20 | Hand/ or hand joint/ or hand palm/ or thenar/ | 46097 |
| 21 | Hand.mp. | 749989 |
| 22 | Hands.mp. | 108197 |
| 23 | Finger/ or index finger/ or little finger/ or middle finger/ or ring finger/ or thumb/ | 55377 |
| 24 | Finger*.mp. | 326947 |
| 25 | Interdigital.mp. | 3530 |
| 26 | Wrist/ | 40590 |
| 27 | Wrist.mp | 82162 |
| 28 | Wrists.mp. | 9630 |
| 29 | Palm*.mp | 160122 |
| 30 | 20 or 21 or 22 or 23 or 24 or 25 or 26 or 27 or 28 or 29 | 1287915 |
| 31 | Child/ or infant/ or preschool child/ or school child/ or toddler/ | 3081241 |
| 32 | Child*.mp. | 3608274 |
| 33 | Infant.mp. | 992271 |
| 34 | Toddler.mp. | 12640 |
| 35 | Juvenile/ or adolescent/ | 2006125 |
| 36 | Juvenile*.mp. | 195427 |
| 37 | Adolescen*.mp. | 2098573 |
| 38 | Teen*.mp. | 52765 |
| 39 | Elementary student/ or high school student/ or middle school student/ | 13797 |
| 40 | elementary student*.mp. | 2498 |
| 41 | High-school student*.mp. | 19409 |
| 42 | Middle-school student*.mp. | 3897 |
| 43 | Day care/ or child day care/ | 14245 |
| 44 | Day care.mp. | 19149 |
| 45 | Daycare.mp. | 2839 |
| 46 | Child care/ or infant care/ or kindergarten/ or nursery/ | 54587 |
| 47 | Child care.mp. | 47816 |
| 48 | Infant care.mp. | 3816 |
| 49 | Kindergarten.mp. | 9378 |
| 50 | Nursery.mp. | 16171 |
| 51 | School/ or high school/ or middle school/ or nursery school/ or primary school/ | 131918 |
| 52 | School*.mp. | 930980 |
| 53 | Pediatrics/ | 103371 |
| 54 | Pediatr*.mp. | 776706 |
| 55 | Paediatr*.mp. | 158109 |
| 56 | Kid.mp. | 4636 |
| 57 | Kids.mp. | 13108 |
| 58 | 31 or 32 or 33 or 34 or 35 or 36 or 37 or 38 or 39 or 40 or 41 or 42 or 43 or 44 or 45 or 46 or 47 or 48 or 49 or 50 or 51 or 52 or 53 or 54 or 55 or 56 or 57 | 5393852 |
| 59 | 19 and 30 and 58 | 4223 |
| 60 | limit 59 to (human and (infant <to one year> or child <unspecified age> or preschool child <1 to 6 years> or school child <7 to 12 years> or adolescent <13 to 17 years>)) | 2877 |

Database(s): **Web of Science Core Collection ALL** 1900 to 26 March 2024 (initial search Aug 27 2022)
Search Strategy:

| # | Searches | Results |
| --- | --- | --- |
| 1 | ALL=(dermatitis* OR dermatos* OR eczema* OR pompholyx OR dyshidro* OR "contact allergy" OR "contact sensitivity" OR pulpitis OR urticaria OR hives ) | 156001 |
| 2 | ALL=(hand OR hands OR finger* OR interdigital OR wrist OR wrists OR palm*) | 1859486 |
| 3 | ALL=(child* OR infant OR toddler OR juvenile* OR adolescen* OR teen* OR "elementary student" OR "high-school student" OR "high school student" OR "middle-school student" OR "middle school student" OR "day care" OR daycare OR "child care" OR "infant care" OR "kindergarten" OR nursery OR school OR pediatr* OR paediatr* OR kid OR kids ) | 18207496 |
| 4 | 1 and 2 and 3 | 2864 |

**Appendix 1b. PRISMA flow diagram for new systematic reviews which included searches of databases and registers only**

**Identification of studies via databases and registers**

Records removed *before screening*:

Duplicate records removed (n = 2125)

Records marked as ineligible by automation tools (n = 0)

Records removed for other reasons (n = 0)

Records identified from:

Databases (n = 8646)

*OVID MEDLINE = 2905*

*EMBASE = 2877*

Web of Science = 2864

**Identification**

Records screened

(n = 6521)

Records excluded

(n = 6093)

Reports sought for retrieval

(n = 458)

Reports not retrieved (unable to retrieve full text)

(n = 7)

**Screening**

Reports excluded:

Wrong patient population (n = 179)

Alternative or confounding diagnosis (n = 33)

Wrong study design (n = 94)

Wrong study outcomes (n = 94)

Wrong setting (n = 2)

Duplicate (n = 2)

Reports assessed for eligibility

(n = 451)

Studies included in review

(n = 47)

Number of Included reports

(n = 62)

**Included**

*From:*  Page MJ, McKenzie JE, Bossuyt PM, Boutron I, Hoffmann TC, Mulrow CD, et al. The PRISMA 2020 statement: an updated guideline for reporting systematic reviews. BMJ 2021;372:n71. doi: 10.1136/bmj.n71
